# The Effects of Physical Activity Timing and Adherence to Physical Activity Guidelines on Sleep in Children With ADHD

**DOI:** 10.1111/sms.70277

**Published:** 2026-04-02

**Authors:** Xiao Liang, Mengping Zhao, Li Su, Justin A. Haegele, Richard H. Xu, Jingjing Li, Andy Choi‐Yeung Tse, Shirley X. Li

**Affiliations:** ^1^ Department of Health and Physical Education The Education University of Hong Kong Hong Kong SAR China; ^2^ Department of Rehabilitation Sciences The Hong Kong Polytechnic University Hong Kong SAR China; ^3^ The Research Institute for Sports Science and Technology The Hong Kong Polytechnic University Hong Kong SAR China; ^4^ School of Public Health Lanzhou University Lanzhou China; ^5^ Department of Human Movement Sciences Old Dominion University Norfolk USA; ^6^ Department of Sports Science and Physical Education The Chinese University of Hong Kong Hong Kong SAR China; ^7^ Department of Psychology University of Hong Kong Hong Kong SAR China

**Keywords:** ADHD, children, physical activity timing, sleep

## Abstract

Regular physical activity (PA) has been shown to benefit sleep. However, the effects of PA timing and adherence to the WHO's recommended 60‐min moderate‐to‐vigorous physical activity (MVPA) guideline on sleep are unclear, especially for children with ADHD who often experience sleep problems. This study examined whether timing of MVPA influenced objectively measured sleep, including sleep latency (SL), sleep efficiency (SE), total sleep time (TST), and wake after sleep onset (WASO), among children with ADHD. This cross‐sectional study involved 253 children with ADHD (Mage = 8.60 ± 1.31 years, 81% boys). MVPA timing was categorized as more than 8 h before bedtime, 3–8 h before bedtime, and less than 3 h before bedtime. Meeting the MVPA guideline was defined as engaging in at least 60 min of MVPA daily. PA and sleep were recorded using an ActiGraph GT9X accelerometer for 7 consecutive days. Among the participants, 174 children (68.8%) met the WHO daily guidelines of 60 min of MVPA. These children had better sleep outcomes than those who did not meet the guideline, with shorter sleep latency, higher sleep efficiency, and less WASO. Regarding PA timing, meeting the MVPA guideline and MVPA time more than 8 h before bedtime were associated with reduced SL, improved SE, and reduced WASO. Conversely, MVPA time less than 3 h before bedtime was associated with decreased SE and increased WASO. Performing MVPA more than 8 h before bedtime and MVPA guideline attainment are crucial for improving sleep outcomes in children with ADHD.

## 1 Introduction

Attention‐deficit/hyperactivity disorder (ADHD) is a common childhood neurodevelopmental disorder characterized by inattention, impulsivity, and hyperactivity. The Diagnostic and Statistical Manual of Mental Disorders, Fifth Edition (DSM‐5) defines three subtypes of ADHD based on symptoms: predominantly hyperactive–impulsive subtype (ADHD‐HI), predominantly inattentive subtype (ADHD‐I), and combined subtype (ADHD‐C) [1]. ADHD has been increasingly recognized as a major global public health concern in young people, with prevalence estimates of 5%–7% in school‐aged children [2].

Healthy sleep is essential for children's physical, psychological, and cognitive functions [3]. Sleep disturbances are often associated with ADHD [4], with up to 59% of children with ADHD experiencing sleep problems [5]. The most common subjective sleep complaints among children with ADHD are sleep latency delay, bedtime resistance, nocturnal awakenings, and daytime sleepiness [6]. Compared with controls without ADHD, children and adolescents with ADHD were found to have longer sleep latency (*g* = 0.399) and lower sleep efficiency (*g* = −0.221), as measured by actigraphy [7].

Similar to sleep, regular physical activity (PA) is well known to benefit the physical and mental health of children with neurodevelopmental disorders, including ADHD [8, 9]. Compelling evidence supports the daily PA‐induced positive effects on improved executive function [10, 11], psychological well‐being [12, 13, 14], and ADHD symptoms [15, 16] in children with ADHD. Meanwhile, PA has also emerged as a promising intervention to improve sleep problems in children with ADHD. For example, a 12‐week PA intervention (3 times per week, 60 min per session) was found to significantly improve sleep latency and decrease sleep disturbances among a group of children with ADHD aged 6–12 years [17]. Notably, the positive effects of PA on sleep quality were maintained for at least 3 months after the intervention [17, 18]. Additionally, a 12‐week jogging intervention resulted in enhanced sleep efficiency, reduced sleep latency, and wake after sleep onset (WASO) [19]. These findings underscore the potential beneficial effects of PA on sleep in children with ADHD.

The *2020 WHO Guidelines on Physical Activity and Sedentary Behaviors* recommend that children and adolescents, including those with disabilities, should engage in at least 60 min of moderate‐to‐vigorous physical activity (MVPA) daily to maintain health. Also, children should perform vigorous‐intensity aerobic activities at least 3 days per week, include muscle‐ and bone‐strengthening activities, and limit sedentary behavior, particularly recreational screen time [20]. Growing evidence shows an association between ADHD and decreased MVPA, with subjective reports indicating that just approximately one‐third of children with ADHD meet the WHO's daily 60‐min MVPA guideline [21]. Moreover, significantly lower MVPA was observed in children with ADHD compared to those without ADHD when accelerometer measured [22]. In addition, children with ADHD with sleep problems were found to spend less time in daily MVPA and be less likely to meet the WHO's 60‐min daily MVPA guideline compared with children with ADHD without sleep problems [5]. However, little is known about whether differences in sleep exist between children with ADHD who meet the recommended MVPA guidelines and those who do not.

Although MVPA guidelines provide recommendations for the frequency, intensity, and duration of daily activity, they do not explicitly articulate a suggestion on the timing of PA (e.g., close to or long before bedtime) [23], which has also been shown to be important for children, especially regarding their sleep. Until recently, common sleep hygiene advice included not engaging in high‐intensity physical activity within 3 h before bedtime, avoiding nighttime sleep disruptions [24] and abstaining from exercising 4–5 h before bedtime to prevent sleep disturbances [25]. These suggestions are based on the belief that exercising too close to bedtime may raise body temperature and physiological arousal, potentially causing a phase delay in the circadian rhythm of sleep, which could affect sleep onset latency and quality [26, 27]. However, recent meta‐analyses have indicated that evening PA generally does not adversely affect sleep, except when vigorous activity ends less than 1 h before bedtime [28]. Additionally, high‐intensity PA performed two to 4 h before bedtime does not appear to disrupt nighttime sleep in young and middle‐aged adults [29]. Given the conflicting results between PA timing and sleep in “normal” sleepers, the existing research is limited on the effects of timing and duration of PA on sleep among populations who often experience sleep problems, such as young people with ADHD.

Therefore, the current study aimed to (a) explore the differences in sleep between children with ADHD who achieve MVPA guideline attainment and those who do not and (b) determine the association among MVPA guideline attainment, the timing of MVPA, and sleep among children with ADHD. Overall, we hypothesized that (a) children with ADHD with MVPA guideline attainment would have better sleep quality and (b) late evening MVPA would be associated with greater impairment in sleep.

## 2 Methods

### 2.1 Participants

A previous study in children with ADHD was used to calculate the sample size (corresponding to an effect size *f* ^*2*^ of 0.128 based on the predictor correlations between MVPA and sleep parameters [10]). Given this effect size, a sample of 98 participants was required to achieve a power of 80% and a level of significance of 5%. Considering the 10% dropout rate, 108 children with ADHD are required in the analysis.

Children clinically diagnosed with ADHD aged 6 to 12 years were recruited in this cross‐sectional study. The clinical diagnosis of ADHD (any subtypes) was confirmed by an expert child psychiatrist based on the Diagnostic and Statistical Manual of Mental Disorders‐Fifth version (DSM‐5) [1]. All participants met the following study criteria: (1) IQ of at least 75 on the Wechsler Intelligence Scale for Children, 4th Edition (WISC‐IV) [30] as assessed by a clinical psychologist; (2) absence of prominent medical conditions that limited PA capability (e.g., asthma and cardiac disease), also checked by pediatricians; and (3) absence of neuropsychiatric disorders (e., bipolar disorder, autism spectrum disorder, and substance abuse) [1]. Participant recruitment was conducted in a children's hospital in Lanzhou, China, from August 2023 to February 2025, and was carried out by the pediatricians following a standard protocol [5]. This study's design followed the Declaration of Helsinki's ethical standards, and the research protocol was approved by the Institutional Review Board of The Hong Kong Polytechnic University (Reference Number: HSEARS20230314005) and Lanzhou University (Reference Number: IRB23071001). Informed consent was obtained from the participants' parents or guardians. Participants individually visited the consulting room with their parents/guardians. Weight and height were measured by clinicians, and body mass index (BMI) was calculated by dividing body mass (kg) by height squared (m2). Then, each participant was asked to wear an Actigraphy on the nondominant wrist, and a logbook was also provided for his/her parents to record put‐on/take‐off time and reasons. After 7 days of wearing it, the participant's parents/guardians brought or mailed the Actigraphy back to the hospital.

### 2.2 Measures

#### 2.2.1 Objectively‐Measured Physical Activity

Physical activity data used for analysis were time spent in MVPA, which was objectively measured using the ActiGraph GT9X Link (ActiGraph, Pensacola, Florida, USA) accelerometer device. All participants were required to wear the device on their nondominant wrist for 7 consecutive days (including 5 weekdays and 2 weekend days) and were instructed to take them off when taking baths or swimming. The sampling rate was set to 60 Hz. Data were analyzed using ActiLife software v6.13.6. The analysis included children with valid data for at least 4 days, with a minimum of 10 h of wear time, and a valid wear period was ≥ 3 valid school days and ≥ 1 valid weekend day [31]. Device and wrist‐specific cut‐points for children represented time spent in MVPA [32]. The Actigraph has been used extensively to measure physical activity and is well‐validated in MVPA records in children with ADHD [33]. Engaging in an average MVPA for ≥ 60 min/day in valid days (e.g., at least 3 school days and 1 weekend day) was considered to meet the WHO‐recommended MVPA guideline [22]. The timing of MVPA was determined based on actigraphy. With a reference to previous research [34, 35], the timing of MVPA was coded as follows: greater than 8 h before bedtime, 3–8 h before bedtime, and less than 3 h before bedtime.

#### 2.2.2 Objectively Measured Sleep

Objectively measured sleep data for analysis consisted of four sleep parameters recorded using the ActiGraph GT9X Link (ActiGraph, Pensacola, Florida, USA) accelerometer. Four sleep parameters, including sleep latency (SL, length of time in minutes from lights out to fall asleep), sleep efficiency (SE, the percentage of actual sleep time divided by the time between sleep onset and sleep offset), total sleep time (TST, total time spent asleep per night), and wake after sleep onset (WASO, length of wake time in minutes between sleep onset and sleep offset), were also measured using an ActiGraph accelerometer. The accelerometer has been widely used to measure sleep in children with and without ADHD [7]. The Sadeh algorithm [36], the most commonly used algorithm for sleep–wake scoring in children, was implemented to identify sleep onset and sleep offset [37].

#### 2.2.3 Sleep Diary

Supplementing the objectively measured sleep, parents/guardians completed a 7‐day sleep diary for the same week when their child with ADHD wore the Actigraph. The daily sleep diary collected information on parent‐reported bedtime and wake‐up time and whether any particular events (e.g., illness and medication) could have disrupted their child's sleep. The specific days for which the sleep diary indicated that actigraphy data were invalid, such as when the Actigraph was removed during sleep, were discarded to ensure optimally reliable data. Additionally, when there was a discrepancy in bedtime and rise time between sleep diary data and actigraphy data (i.e., a difference greater than 30 min) on a particular night, the data for this night were not included in the analysis.

### 2.3 Data Analysis

All statistical analyses were performed using the Statistical Package for the Social Sciences (SPSS, version 29.0). Children with ADHD were divided into two groups: active children (who attained the WHO 60‐min of MVPA guideline) and inactive children (who did not attain the WHO 60‐min of MVPA guideline). Independent *t*‐tests or chi‐square tests were used to compare the differences in demographic and clinical characteristics (e.g., age, sex, IQ, height, weight, BMI, ADHD subtype, comorbidity, and medication) between the two groups. Then, a multivariate analysis of covariance (ANCOVA) controlling for age, sex, IQ, BMI, comorbidity, medication, and ADHD subtypes was carried out to compare the group differences in PA levels, timing of MVPA, and sleep parameters. The effect size was reported as an η2 (eta‐squared) value for ANCOVA. η2 = 0.01 indicates a small effect, η2 = 0.06 indicates a medium effect, and η2 = 0.14 indicates a large effect [38]. Bivariate correlations were calculated among the variables of interest: timing of MVPA (i.e., 3 h before bedtime; 3–8 h before bedtime; 8 h before bedtime), MVPA guideline attainment, and sleep parameters (i.e., SL, SE, TST, and WASO). Lastly, hierarchical regression models were used to examine the association of sleep parameters (i.e., SL, SE, TST, and WASO) with the timing of MVPA. For all the analyses, statistical significance was set at *p* < 0.05.

## 3 Results

### 3.1 Characteristics of the Participants

Table 1 shows the characteristics of the participants. Of the initially recruited 270 children with ADHD, 17 participants were excluded from data analyses due to less than 4 days of valid PA/sleep data, resulting in a final dataset of 253 children with ADHD. All included participants had valid accelerometer data for at least 4 days, including 3 weekdays and 1 weekend day. Among the 253 participants with ADHD in this study, 205 (81%) were boys, while 48 (19%) were girls. The average age of the participants was 8.60 ± 1.31 years old. The mean IQ of the participants was 92.69 ± 10.62. Regarding the ADHD subtype, the majority of the participants were diagnosed with ADHD Inattentive subtype (ADHD‐I) (*n* = 140, 55.3%), followed by ADHD Combined subtype (ADHD‐C) (*n* = 86, 34.0%) and the ADHD Hyperactive–Impulsive subtype (ADHD‐H) (*n* = 27, 10.7%). In addition, 25.7% of the participants were diagnosed as having comorbid oppositional defiant disorder (ODD) and 16.6% reported regular use of medication for ADHD management. Based on the WHO 60‐min of MVPA guideline, the active children group consisted of 174 participants with ADHD (68.8%) (M_age_ = 8.51, 83.9% boys), and the inactive children group consisted of 79 participants with ADHD (31.2%) (M_age_ = 8.81, 74.7% boys). There were no significant differences in age, sex, IQ, BMI, ADHD subtype, comorbidity, and medication between active and inactive children.

**TABLE 1:** Descriptive statistics in ADHD groups (*n* = 253).

Variable	Total (*n* = 253) (95% CI)	Active children (95% CI) (*n* = 174, 68.8%)	Inactive children (95% CI) (*n* = 79, 31.2%)	*p*
Sex (*n*, % male)	205, 81.0%	146, 83.9%	59, 74.7%	0.083
Age (years)	8.60 (8.44, 8.76)	8.51 (8.32, 8.69)	8.81 (8.49, 9.12)	0.091
IQ	92.69 (91.38, 94.01)	93.32 (91.78, 94.86)	91.30 (88.79, 93.81)	0.162
Weight (kg)	30.52 (29.28, 31.76)	29.39 (28.17, 30.61)	33.00 (30.11, 35.91)	0.008*
Height (cm)	132.93 (131.74, 134.12)	131.73 (130.49, 132.98)	135.55 (132.95, 138.15)	0.003*
BMI (kg/m^2^)	16.92 (16.50, 17.34)	16.70 (16.25, 17.16)	17.40 (16.49, 18.30)	0.132
ADHD subtype (*n*, %)				
Inattention	140, 55.3%	91, 52.3%	49, 62.0%	0.142
Hyperactivity	27, 10.7%	17, 9.8%	10, 12.7%	
Combined	86, 34.0%	66, 37.9%	20, 25.3%	
Comorbidity (*n*, %)				
Oppositional defiant disorder	65, 25.7%	44, 25.3%	21, 26.6%	0.244
Anxiety	21, 8.3%	10, 5.7%	11, 13.9%	
Tic disorder	3, 1.2%	3, 1.7%	0	
Obsessive‐compulsive disorder	1, 0.3%	1, 0.5%	0	
Medication (*n*, %)	42 (16.6%)	22, 14.3%	20, 8.1%	0.256
Concerta	8	3	5	
Strattera	10	6	4	
Xiao'er Zhili Syrup (Kuihua)	13	5	8	
Jingling Oral Liquid	8	5	3	
Clonidine	3	3	0	

Abbreviations: BMI: Body mass index; IQ: Intelligence quotient.

### 3.2 Comparisons of Physical Activity Levels, Timing of MVPA, and Sleep Parameters Between Active and Inactive Children

Table 2 presents the group comparison of all outcome variables. Compared to active children, inactive children with ADHD spent significantly less time in MVPA daily (F = 215.67, η2 = 0.469, *p* < 0.001) and in three defined MVPA timing slots, including less than 3 h before bedtime (F = 39.82, *η* ^*2*^ = 0.140, *p* < 0.001), 3–8 h before bedtime (F = 137.19, *η* ^*2*^ = 0.360, *p* < 0.001), and greater than 8 h before bedtime (F = 141.71, *η* ^*2*^ = 0.367, *p* < 0.001). Inactive children with ADHD had longer sleep latency (F = 50.98, η2 = 0.173, *p* < 0.001), lower sleep efficiency (F = 24.04, η2 = 0.090, *p* < 0.001), and longer WASO (F = 17.54, η2 = 0.067, *p* < 0.001), but similar TST (F = 0.805, *p* = 0.370), indicating poorer sleep quality, compared to those with MVPA guideline attainment. In addition, there was no significant difference in sleep–wake patterns (i.e., bedtime and rise time) between active and inactive children with ADHD.

**TABLE 2:** Comparisons of physical activity levels, timing of exercise, and sleep parameters between active and inactive children with ADHD.

	Active children (*n* = 174, 68.8%) M ± SD	Inactive children (*n* = 79, 31.2%) M ± SD	*F*	*p*	*η2*
Physical activity levels					
MVPA (mins)	90.39 ± 26.46	42.37 ± 11.08	215.67	< 0.001	0.469
Weekly MVPA Guideline Adherence Rates					
Proportion of days meeting MVPA guideline (%)	77.94 ± 21.84	21.25 ± 22.94	320.35	< 0.001	0.568
Timing of MVPA (mins)					
Less than 3 h before bedtime	14.57 ± 9.23	7.46 ± 4.80	39.82	< 0.001	0.140
3–8 h before bedtime	34.74 ± 12.93	15.60 ± 5.80	137.19	< 0.001	0.360
Greater than 8 h before bedtime	41.02 ± 14.83	19.30 ± 6.38	141.71	< 0.001	0.367
Sleep parameters (actigraphy)					
Sleep latency (mins)	21.94 ± 8.99	33.48 ± 15.20	50.98	< 0.001	0.173
Sleep efficiency (%)	76.66 ± 6.47	71.87 ± 8.04	24.04	< 0.001	0.090
Total sleep time (mins)	404.17 ± 33.91	397.32 ± 44.20	0.805	0.370	0.003
Wake after sleep onset (mins)	102.38 ± 36.87	124.24 ± 45.50	17.54	< 0.001	0.067
Sleep–wake pattern (actigraphy)					
Bedtime (hh:mm)	22:10	21:50	0.376	0.540	0.002
Rise‐time (hh:mm)	07:18	07:26	3.518	0.062	0.013

*Note:* MVPA guideline attainment cutoff of < 60 min/day indicating “inactive children”; MVPA guideline attainment cutoff of ≥ 60 min/day indicating “active children”; MVPA, moderate‐to‐vigorous physical activity; BMI, body mass index; M, mean; SD, standard deviation; P, *P*‐value; Weekly MVPA guideline adherence rates: The proportion of days during the actigraphy monitoring week on which participants met the MVPA guideline; Group comparison results controlling age, sex, BMI, IQ, comorbidity, medication, and ADHD subtype.

### 3.3 Association of Timing of MVPA and MVPA Guideline Attainment (IV) With Sleep Parameters (DV)

Table 3 presents the correlations among the timing of MVPA (i.e., 3 h before bedtime; 3–8 h before bedtime; 8 h before bedtime), MVPA guideline attainment, and sleep parameters (i.e., SL, SE, TST, and WASO). The results of skewness (|skewness| < 3) and kurtosis (|kurtosis| < 10) tests showed that data were normally distributed [39]. MVPA less than 3 h before bedtime was negatively associated with sleep latency. MVPA at 3–8 h before bedtime was negatively correlated with sleep latency but was positively related to efficiency. MVPA at greater than 8 h before bedtime and meeting MVPA guideline were negatively correlated with sleep latency and WASO but were positively associated with sleep efficiency.

**TABLE 3:** Association of timing of exercise and MVPA guideline attainment (IV) with sleep parameters (DV) (r‐values).

	1	2	3	4	5	6	7	8
1. MVPA at < 3 h before bedtime (mins)	—							
2. MVPA at 3–8 h before bedtime (mins)	0.341**	—						
3. MVPA > 8 h before bedtime (mins)	283**	0.653**	—					
4. MVPA guideline attainment (yes/no)	378**	0.622**	0.619**	—				
5. Sleep latency (mins)	−0.143**	−0.273**	−0.390**	−0.429**	—			
6. Sleep efficiency (%)	−0.72	0.142*	0.289**	0.303**	−0.437**	—		
7. Total sleep time (mins)	−0.005	0.052	−0.001	0.085	−0.148*	671**	—	
8. Wake after sleep onset (mins)	0.122	−0.093	−0.253**	−0.248**	0.215*	−0.945**	−0.517**	—
Mean	12.35	28.76	34.24	—	25.54	75.17	402.03	109.20
SD	8.75	14.29	16.29	—	12.48	7.33	37.37	40.95

Abbreviations: *N* = 253. Moderate‐to‐vigorous physical activity. For MVPA guideline attainment, 0 = without MVPA guideline attainment, 1 = with MVPA guideline attainment.

* *p* < 0.05.

** *p* < 0.01.

### 3.4 Association of MVPA Guideline Attainment and Timing of MVPA With Sleep Parameters

A three‐step hierarchical regression analysis was carried out to identify significant factors associated with sleep parameters such as sleep latency, sleep efficiency, and WASO. As the MVPA attainment guidelines and timing of MVPA were not correlated with total sleep time, we excluded the regression analysis with total sleep time. The independent variables were entered as follows: Step 1 included covariates such as IQ, age, BMI, sex, ADHD subtype, comorbidity and medication use; Step 2 included MVPA guideline attainment; and Step 3 included the timing of MVPA such as less than 3 h before bedtime, 3–8 h before bedtime, and greater than 8 h before bedtime.

The results of the regression analysis related to the variables associated with sleep parameters are depicted in Table 4. Regarding sleep latency, we found that MVPA guideline attainment (*β* = −0.318, *p* < 0.001) and MVPA at greater than 8 h before bedtime (*β* = −0.265, *p* = 0.001) made a significant contribution and accounted for 23.2% of the variance of the sleep latency. Regarding sleep efficiency, we found MVPA guideline attainment (*β* = 0.301, *p* < 0.001), MVPA at less than 3 h before bedtime (*β* = −0.211, *p* = 0.002), and MVPA at greater than 8 h before bedtime (*β* = 0.268, *p* = 0.002) significantly contributed to the quality of sleep efficiency. Regarding WASO, we found that MVPA guideline attainment (*β* = −0.276, *p* = 0.001), MVPA at less than 3 h before bedtime (*β* = 0.231, *p* < 0.001), and MVPA at greater than 8 h before bedtime (*β* = −0.263, *p* = 0.002) significantly contributed to predicting the duration of WASO.

**TABLE 4:** Association of MVPA guideline attainment and timing of exercise with sleep.

Predictor Variables	Sleep latency			Sleep efficiency			Wake after sleep onset		
	Model 1	Model 2	Model 3	Model 1	Model 2	Model 3	Model 1	Model 2	Model 3
Step 1: Demographic									
IQ	−0.119	−0.085	−0.094	0.135*	0.111	0.101	−0.114	−0.093	−0.082
Age	0.008	−0.020	−0.023	−0.040	−0.020	−0.025	−0.007	−0.024	−0.018
BMI	0.036	0.003	0.016	−0.030	−0.006	−0.045	−0.034	−0.054	−0.013
Sex	0.042	−0.004	−0.029	−0.023	0.010	0.040	0.027	−0.001	−0.031
ADHD‐HI subtype	0.055	0.040	0.049	−0.122	−0.112	−0.091	0.135*	0.127	0.102
ADHD‐C subtype	−0.033	0.012	0.016	−0.056	−0.088	−0.101	0.075	0.103	0.117
Comorbidity	0.034	0.002	−0.020	−0.003	0.019	0.028	0.016	−0.003	−0.009
Medication use	−0.094	−0.070	−0.080	0.029	0.012	0.028	0.006	0.021	0.004
Step 2: Physical activity									
MVPA guideline attainment		−0.419**	−0.318**		0.298**	0.301**		−0.056**	−0.276**
Step 3: Timing of exercise									
3 h before bedtime			0.012			−0.211**			0.231**
3‐8 h before bedtime			0.092			−0.140			0.151
8 h before bedtime			−0.265**			0.268**			−0.263**
*R* ^2^	0.033	0.198	0.232	0.034	0.118	0.434	0.036	0.098	0.176
*F*	1.026	6.650	6.032	1.070	3.603	4.653	1.142	2.934	4.275
Δ*R* (*R* ^2^ change)	0.033	0.165	0.034	0.034	0.084	0.071	0.036	0.062	0.078
Δ*F* (*F* change)	1.026	49.997	3.550	1.070	23.095	7.000	1.142	16.687	7.581

Abbreviations: BMI: body mass index; MVPA: moderate‐to‐vigorous physical activity Coefficients shown are standardized beta coefficients; *N* = 253. For Sex, boy = 0, girl = 1. For ADHD subtype, ADHD‐I = 0, ADHD‐HI/ADHD‐C = 1. For Comorbidity, no comorbid disorders =0, comorbid disorders = 1. For medication use, medication‐naïve = 0, medicated = 1. For MVPA guideline attainment, without MVPA guideline attainment = 0, meeting MVPA guideline attainment = 1.

* *p* < 0.05.

** *p* < 0.01.

## 4 Discussion

This study aimed to compare the differences in sleep parameters and the timing of MVPA between children with ADHD who attain MVPA guidelines and children with ADHD who do not attain MVPA guidelines and to determine the relationship between the timing of MVPA and sleep in children with ADHD.

The most important and novel finding was that meeting the WHO‐recommended 60‐min MVPA guideline was negatively related to sleep latency and WASO and positively correlated with sleep efficiency among children with ADHD. Consistent with previous studies showing that MVPA was positively related to sleep among adolescents with ADHD [40] and more than 60 min per PA intervention significantly improved sleep in children and adolescents with neurodevelopmental disorders [35], our results strengthen earlier findings that PA programs with MVPA intensity may have beneficial effects on improving sleep problems in children with ADHD [18]. Among our participants, 68.8% met the WHO‐recommended 60 min of MVPA guidelines. Children with ADHD who attain the MVPA guideline show better sleep quality than those who do not meet the MVPA guideline, exhibiting shorter sleep latency and WASO, higher sleep efficiency, and longer total sleep time. These findings highlight the importance of promoting sleep quality in children with ADHD.

Overall, we found that children with ADHD engaged in an average of 75.35 min of MVPA per day in the current study. It was noted that previous findings on accelerometer‐measured MVPA on children with ADHD are mixed in different regions, such as Taiwan (51.75 min/day) [15], Germany (38.47 min/day) [11], the USA (23.5 mins/day) [41], and Mainland China (79.06 mins/day) [14]. Thus, disparities in MVPA among children with ADHD warrant further investigation concerning potential sample characteristics or cultural factors. The *2020 WHO Guidelines on Physical Activity and Sedentary Behaviors* recommended that MVPA can improve impaired cognition, including attention, executive function, and social disorders in children with ADHD [20]. Longitudinal studies identifying the daily MVPA threshold are much needed for children with ADHD to uphold healthy behaviors.

The key finding of this study was that MVPA at 8 h before bedtime was negatively related to sleep latency and WASO and positively associated with sleep efficiency. Consistent with a recent randomized controlled trial (RCT), a 12‐week morning jogging intervention significantly improved sleep efficiency, sleep onset latency, and WASO in children with ADHD [19]. Furthermore, an experimental study showed that 30 min exercising at 11:00 AM daily with sun exposure significantly improved sleep quality [42]. The possible explanation is that daylight entering the retina during morning exercise triggers signals to the brain's suprachiasmatic nucleus (SCN), which functions as the body's master clock. The SCN coordinates various physiological processes, including regulating the sleep–wake cycle. In response to daylight exposure, the SCN directs the pineal gland to decrease melatonin secretion. Reduced melatonin levels during the day contribute to maintaining alertness and wakefulness. Morning exercise and exposure to natural light help synchronize the body's internal clock with the external environment, strengthening the circadian rhythm. This synchronization ensures that melatonin production increases in the evening as daylight diminishes, facilitating sleep onset [43, 44]. However, the latest meta‐analysis showed that morning PA intervention showed nonsignificant effects on sleep in children and adolescents with neurodevelopmental disorders [35]. Due to the limited studies included in the analysis (*n* = 3), further evidence is required to elucidate the relationship between morning exercise and sleep in children with ADHD.

The novel findings of this study showed that MVPA at 3 h before bedtime was positively correlated with WASO and negatively related to sleep efficiency. Previous meta‐analyses showed that acute evening exercise within 4 h of bedtime did not disrupt nighttime sleep in healthy young and middle‐aged adults [28, 29]. This may be because exercising too close to bedtime can lead to physiological arousal, negatively impacting sleep. This arousal can manifest as a sustained increase in core body temperature, elevated heart rate, and heightened sympathetic nervous system activation. These physiological changes can interfere with falling asleep and a potential change in circadian rhythms [27, 45, 46]. For individuals with ADHD, these effects may be more pronounced due to existing challenges in regulating autonomic functioning [47]. ADHD is often comorbid with other conditions, including sleep disorders, and disturbances in autonomic arousal can exacerbate these issues [48]. Evening exercise might further complicate the regulation of autonomic functions in individuals with ADHD, potentially leading to increased sleep difficulties. PA plays a crucial role in maintaining a healthy sleep–wake cycle and improving sleep quality, thereby promoting more restorative rest [49]. Exercise helps regulate endogenous circadian rhythms and has been shown to boost melatonin levels, particularly in children with neurodevelopmental disorders, including ADHD [35]. Melatonin deficiency is linked to sleep problems, including delayed sleep onset and early waking. Evidence suggests that targeted PA interventions can effectively modulate melatonin secretion in this group, thereby improving sleep quality by increasing melatonin production [50]. Therefore, it may be beneficial for individuals, particularly children with ADHD, to schedule exercise earlier in the day to minimize its impact on sleep. This approach can help ensure that the body has adequate time to return to a state conducive to restful sleep.

To the best of our knowledge, this study was the first to explore the timing of MVPA and adherence to the MVPA guideline on sleep in children with ADHD. A primary strength of this study was using actigraphy to objectively and prospectively measure physical activity and sleep in children with ADHD in their naturalistic environment, which was not fully considered or adopted in previous research. Nonetheless, this study had some limitations. First, we used a cross‐sectional design, which limits the possibility of exploring the causal relationships between the timing of exercise and sleep in children with ADHD. Further research with an experimental design is needed to determine the best timing of exercise for improving sleep in children with ADHD. Also, a longitudinal study design is recommended as it would enable tracking changes over time and offer stronger evidence regarding whether MVPA timing influences sleep patterns or vice versa. Second, we did not perform a clinical interview to ascertain presence of sleep disorder in the recruited participants. Further studies should consider using objective and reliable measures of sleep (e.g., polysomnography) to understand the effects of sleep in children with ADHD comprehensively. Third, controls without ADHD were not recruited for the current study. Fourthly, our study did not specifically examine vigorous‐intensity activities in children with ADHD. Further research is recommended to investigate the dose–response relationship between daily vigorous‐intensity activities and sleep, as well as other health indicators for children with ADHD [51]. Fifthly, we recognize that the compositional nature of 24‐h activity data, where time allocated to sleep, physical activity, and sedentary behavior (screen time) sums to a fixed total, is a limitation of our study as the fixed‐sum constraint can influence associations between sleep duration and MVPA. Further studies should consider exploring the relationships between 24‐h activity and other health‐related outcomes in children with ADHD. Lastly, we acknowledge that there are no established, specific intensity thresholds for children with ADHD. We employed wrist‐specific cut‐points developed for children with typical development, which may overestimate MVPA and potentially lead to a higher proportion of participants being classified as meeting the guidelines. Future studies should also evaluate the impact of the chosen accelerometer cut‐off points on MVPA estimation.

As such, it is recommended that regular participation in MVPA, especially in the morning time slots, is beneficial for improving sleep in children with ADHD. Additionally, school‐based PA interventions during the daytime are encouraged for children with ADHD, which can help researchers explore the mechanisms underlying exercise‐induced sleep improvements and determine the optimal timing for performing MVPA to enhance sleep in children with ADHD and those with sleep disturbances.

## 5 Perspective

The current study provides evidence that children with ADHD who have achieved the MVPA guideline have better sleep quality than those who have not. Attainment of the MVPA guideline and MVPA at 8 h before bedtime are associated with improved sleep. MVPA close to bedtime is related to decreased sleep efficiency and increased WASO. Although our study did not determine the direction of the causal relationship between exercise timing and sleep, the observed association emphasizes the potential importance of MVPA at 8 h before bedtime as a key factor in improving sleep among children with ADHD. Future longitudinal and interventional studies are needed to identify the optimal timing of exercise for sleep improvement in children with ADHD and other clinical groups, such as children with autism spectrum disorder who also face sleep disturbances.

## Author Contributions

Xiao Liang: Conceptualization, Data collection, Formal analysis, Funding acquisition, Writing – original draft. Mengping, Zhao, Li Su: Data collection, Formal analysis. Richard H. Xu, Justin A. Haegele, Jingjing Li, Andy Choi‐Yeung Tse: Data analysis, Writing – review and editing; Shirley X. Li: Supervision, Formal analysis, Writing – review and editing.

## Funding

This work was supported by the Research Institute for Sports Science and Technology, The Hong Kong Polytechnic University [P0050100] and the Young Collaborative Research Grant from Research Grants Council, Hong Kong SAR (Ref. C7005‐24Y).

## Conflicts of Interest

The authors declare no conflicts of interest.

## Data Availability

The data that support the findings of this study are available from the corresponding author upon reasonable request.
